# Radiomics: a critical step towards integrated healthcare

**DOI:** 10.1007/s13244-018-0669-3

**Published:** 2018-11-12

**Authors:** Zuhir Bodalal, Stefano Trebeschi, Regina Beets-Tan

**Affiliations:** 1 Department of Radiology, The Netherlands Cancer Institute, Amsterdam, The Netherlands; 2 GROW School for Oncology and Developmental Biology, Maastricht University, Maastricht, The Netherlands

**Keywords:** Quantitative imaging, Radiomics, Deep learning, Healthcare systems, Integrated systems

## Abstract

**Abstract:**

Medical imaging is a vital part of the clinical decision-making process, especially in an oncological setting. Radiology has experienced a great wave of change, and the advent of quantitative imaging has provided a unique opportunity to analyse patient images objectively. Leveraging radiomics and deep learning, there is increased potential for synergy between physicians and computer networks—via computer-aided diagnosis (CAD), computer-aided prediction of response (CARP), and computer-aided biological profiling (CABP). The ongoing digitalization of other specialties further opens the door for even greater multidisciplinary integration. We envision the development of an integrated system composed of an aggregation of sub-systems interoperating with the aim of achieving an overarching functionality (in this case‚ better CAD, CARP, and CABP). This will require close multidisciplinary cooperation among the clinicians, biomedical scientists, and (bio)engineers as well as an administrative framework where the departments will operate not in isolation but in successful harmony.

**Key Points:**

• *The advent of quantitative imaging provides a unique opportunity to analyse patient images objectively.*

• *Radiomics and deep learning allow for a more detailed overview of the tumour (*i.e., *CAD, CARP, and CABP) from many different perspectives.*

• *As it currently stands, different medical disciplines have developed different stratification methods, primarily based on their own field—often to the exclusion of other departments.*

• *The digitalization of other specialties further opens the door for multidisciplinary integration.*

• *The long-term vision for precision medicine should focus on the development of integration strategies, wherein data derived from the patients themselves (*via *multiple disciplines) can be used to guide clinical decisions.*

Medical imaging has historically played a key role in cancer screening, diagnosis, staging, and therapeutic response monitoring. On a daily basis, treating physicians rely on input from imaging to help formulate patient management plans [1]. This is especially true within the context of modern oncological guidelines, where patients are stratified into increasingly complex subgroups based on biological, clinical, and radiological parameters.

Historically, qualitative semantic features were used to describe tumour morphology—as observed in the patient image. These descriptions were a reflection of a scoring system based on visual assessment. Semantic features were shown in the literature to have correlations with stage, prognosis, and even response prediction [2]. However, as one could imagine, this method suffered from shortcomings rooted in its dependence on subjective scoring and the limited sensitivity of the human eye.

Ubiquitous modern imaging modalities, such as CT, MRI, and PET in radiology (and digital images in pathology), are primarily quantitative in nature. This characteristic is harnessed, using computational algorithms to extract quantitative features and generate mineable data. Rather than relying solely on subjective interpretation of images, these quantitative features can be used to objectively characterize tumour morphology.

In radiomics, medical images are processed to generate quantitative features and these mineable data can then be used for clinical purposes. Radiomic features serve the purpose of describing morphological characteristics (e.g. density distribution, recurrent patterns and textures, shape and outline, etc.) in an objective, quantitative manner. The ambition is to find completely non-invasive radiomic features that can be used as predictive and prognostic biomarkers.

## The promise of radiomics

**Fig. 1:**
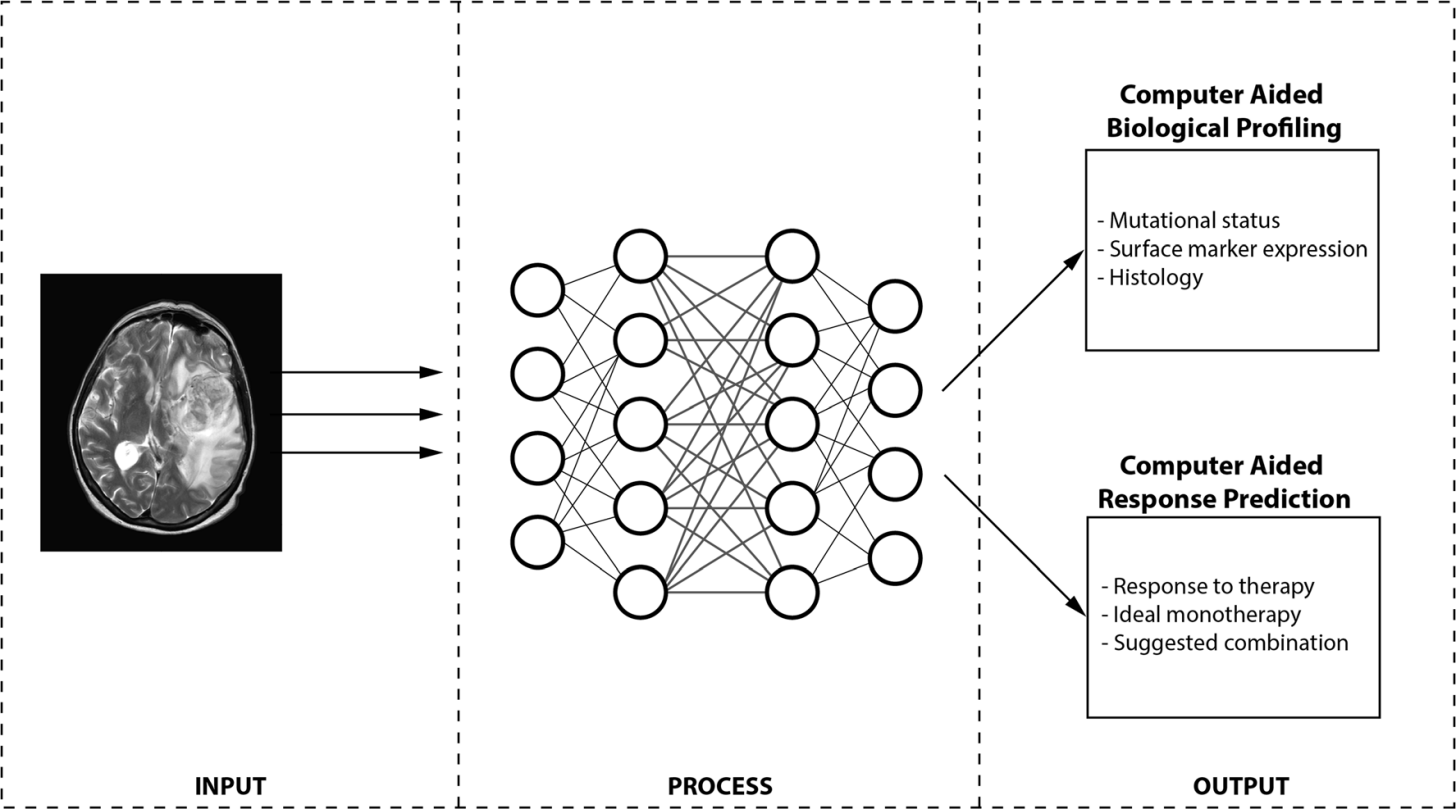
A schematic of a future radiomics pipeline highlighting a simplified workflow for CABP and CARP wherein patient images are input into a specialized (series of) AI algorithm(s) and, based on the outcome, can be classified. CABP algorithms assess the profile of the tumour (for stratification) while CARP algorithms focus purely on the prediction of response to (and ultimately selection of) therapy. CAD: computer-aided diagnosis; CARP: computer-aided response prediction; CABP: computer-aided biological profiling

The advent of radiomics has opened a brand new avenue in cancer research and presents a unique opportunity to data scientists and radiologists alike. Broadly speaking, two prominent potentials have emerged for radiomics—tumour characterization and therapeutic response prediction (Fig. 1).

The search has begun to identify imaging markers to be used to assess biological parameters (i.e. *genetic mutations or surface expression of particular molecules*) in the tumour. Normally, such biological assessment of a tumour is achieved by biopsy—a process that is highly invasive, carries potential risk for patient morbidity, and can only elucidate information for lesions in sites easily accessible to surgeons. Radiomics provides the opportunity to non-invasively assess the biological profile (i.e. surface marker expression, genetic mutational status, blood markers, etc.) of all the lesions simultaneously and instantaneously. With the increased use of computer models to diagnose conditions and predict response to therapy, this new field where biological parameters can non-invasively be assessed using quantitative features and computer models can be termed computer-aided biological profiling (CABP).

One of the earlier studies to leverage radiomic features in the assessment of genetic mutational status (i.e. radiogenomics) was the work of Segal et al. in human liver cancer where combinations of 28 imaging traits were shown to be capable of reconstructing 78% of the global gene expression profile (i.e. mRNA levels) of these tumours [3]. Further research ensued on a number of tumour types—with varying degrees of success.

With the rise of deep-learning-based image analysis, computer algorithms can be used to extract radiomic features on a large scale that could then be linked to predictive and prognostic biomarkers in cancer (that would otherwise be obtained surgically).

Unlike more traditional radiomics approaches where feature extraction and data analysis consisted of two separate steps, deep learning fuses these processes together and iteratively optimizes one with respect to the other. In other words, deep learning provides radiomics models with optimal features and optimal data analysis for a specific clinical problem. This advanced form of computer-aided biological profiling (where a neural network can extract features and link them together on a massive scale) can be termed deep learning mediated tumour profiling (DL-TP).

The next application of radiomic features in cancer research was prediction of response to different forms of treatment [i.e. computer-aided response prediction (CARP)]. In non-small-cell lung cancer (NSCLC), Coroller et al. identified seven features that were predictive for pathological gross residual disease and one feature for pathological complete response [4]. Further studies later identified other radiomic features that would predict the response to conventional treatment (i.e. chemo-/radiotherapy) in bladder cancer [5] and locally advanced rectal cancer [6].

## Integrated systems in healthcare

While the suffix “-omics” has come to denote the idea of extracting valuable information from data sets, radiomics is only the latest addition to the ever-growing list of new fields of study within the fusion of advanced technology and modern medicine. Images derived from tissue (e.g. general microscopy, immunohistochemistry, etc.) have also been subject to quantitative analysis and new information is being generated beyond what would be observed by a pathologist, i.e. pathomics. Genomics, the branch of molecular biology concerned with the mapping of the human genome, has helped to identify many genetic mutations and pathways that have been used for prognostication or as novel targets for modern therapeutics and has heavily relied on computer models developed by skilled bioinformaticians.

As it currently stands, different medical disciplines have developed different stratification methods, primarily based on their own field (i.e. radiological classifications, pathological and chemical laboratory classifications, clinical checklists used for prognostication, etc.)—quite often to the exclusion of other departments. As these traditional scoring systems were often based on subjective interpretations of analogue readouts, combining these disparate outputs is quite challenging. The rise of the quantitative aspects of various medical disciples (i.e. the “-omics”) presents a remarkably unique opportunity wherein information from different diagnostic modalities can be objectively integrated.

## Conclusion

**Fig. 2:**
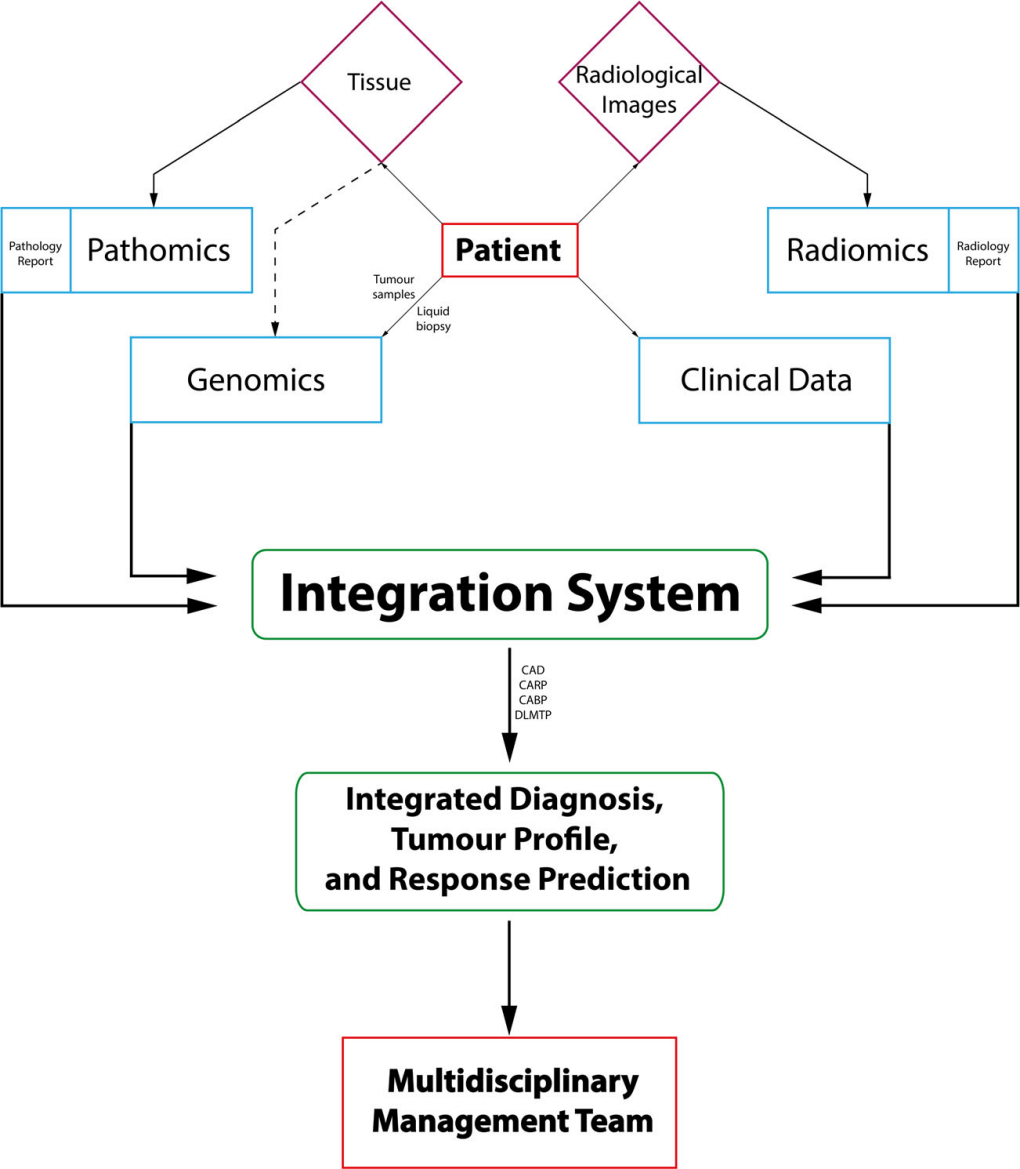
A schematic flow chart envisioning the usage of patient-derived data (in light blue) from raw materials (in purple) as a means to improve CAD, CARP, and CABP and ultimately help guide decisions by the multidisciplinary management team

The long-term vision for precision medicine should focus on the development of integration strategies, wherein data derived from the patients themselves can be used to guide the treating physician. Through intricate analyses that integrate clinical data, blood markers, pathomics, radiomics, and genomics, we envision that a patient can be provisionally diagnosed (via computer-aided diagnosis), stratified into a molecular subtype of their tumour (via computer-aided biological profiling), and have a recommended treatment formulated (via computer-aided response prediction). This aggregation of sub-systems cooperating with the aim of achieving an overarching functionality (in this case‚ better CAD, CARP, and CABP) is termed the integration system (see Fig. 2). This will require hand-in-hand multidisciplinary collaboration between the biomedical field (i.e. clinicians, geneticists, radiologists, pathologists, clinical chemists) and the technical field (i.e. computer scientists, physicists, engineers, statisticians, and mathematicians) as well as an organizational structure wherein the departments will operate not in isolation but in successful integration.
